# Phrenic nerve stimulation enhances upper airway patency during drug-induced sleep endoscopy in obstructive sleep apnea

**DOI:** 10.1093/annalsats/aaoag079

**Published:** 2026-04-26

**Authors:** Jason L Yu, Katherine P Gouldman, Bailey L Kane, Kaitlin N Manning, Margaret C McEachran, David Bourn, Hartmut Schneider, Alan R Schwartz

**Affiliations:** Department of Otolaryngology-Head and Neck Surgery, Emory University, Atlanta, Georgia; Department of Otolaryngology-Head and Neck Surgery, Emory University, Atlanta, Georgia; Department of Otolaryngology-Head and Neck Surgery, Emory University, Atlanta, Georgia; Clinical Research Department, Lunair Medical Inc, Minneapolis, MN, United States; Clinical Research Department, Lunair Medical Inc, Minneapolis, MN, United States; Clinical Research Department, Lunair Medical Inc, Minneapolis, MN, United States; Division of Pulmonary Medicine, Philipps University, Marburg, Germany; Department of Otorhinolaryngology-Head and Neck Surgery, University of Pennsylvania Perelman School of Medicine, Philadelphia, PA, United States; Department of Otolaryngology, Vanderbilt University School of Medicine, Nashville, TN, United States

**Keywords:** neurostimulation, transcutaneous nerve stimulation, diaphragm, pharyngeal muscles

## Abstract

**Rationale:**

Obstructive sleep apnea (OSA) is characterized by recurrent upper airway (UA) collapse during sleep. Continuous positive airway pressure (CPAP) is an effective but often poorly tolerated treatment. Neurostimulation targeting UA dilators has emerged as an alternative therapy, though coordinating stimulation of multiple effector muscles may be challenging. Phrenic nerve stimulation (PNS) offers a potential alternative that may engage both efferent and afferent respiratory mechanisms to enhance airway patency.

**Objective:**

To examine how PNS timing affects UA patency in OSA patients undergoing drug-induced sleep endoscopy.

**Methods:**

Obstructive sleep apnea patients underwent drug-induced sleep endoscopy. Subtherapeutic CPAP was applied to maintain stable flow-limited breathing for PNS experiments. Transcutaneous PNS was delivered via a cervical electrode at different phases of respiration: end-expiration to inspiration (E→I), inspiration only (I→I), and mid-inspiration to expiration (I→E). Sequential stimulations were also performed at E→I to observe sustained effects. Effect of stimulation on inspiratory flow (V_I_), tidal volume (TV), and minute ventilation (MV) were quantified using mixed-effects models.

**Measurements and Main Results:**

A total of 19 patients participated: 13 completed both isolated and sequential protocols, and 6 completed only the sequential protocol. Isolated PNS timed to E→I increased V_I_ (64%) and TV (71%) compared with baseline. Sequential PNS at E→I produced sustained improvements with further increases in V_I_ (171%), TV (173%), and MV (169%).

**Conclusions:**

Phrenic nerve stimulation delivered at end-expiration significantly improved UA patency and ventilation, with sequential stimulation further potentiating these effects. Phase-locked PNS responses implicate underlying neural afferent mechanisms and suggest novel PNS strategies for treating OSA.

## Introduction

Obstructive sleep apnea (OSA) is a sleep-related breathing disorder marked by recurrent upper airway (UA) narrowing and collapse, leading to intermittent hypoxia and sleep fragmentation.^1^^,^^2^ Obstructive sleep apnea is associated with increased risk of cardiovascular disease, metabolic dysfunction, neurocognitive impairment, and reduced quality of life.^1^^,^^2^ While continuous positive airway pressure (CPAP) remains the gold standard treatment, up to 40% of patients are unable to tolerate it, underscoring the need for alternative therapies.^3^

Neurostimulation of UA dilator muscles has emerged as a new strategy for treating OSA. Hypoglossal nerve stimulation (HGNS), which activates the genioglossus muscle to dilate the pharynx, can effectively treat OSA.^4^ However, HGNS is limited by stringent selection criteria and variable response rates, with only 60%-80% of carefully selected patients achieving clinical response.^4^^,^^5^ More recently, ansa cervicalis neurostimulation, which activates the laryngeal strap muscles to caudally displace the pharynx, has been shown to further improve UA collapsibility when combined with HGNS.^6^ These findings highlight the therapeutic potential of neurostimulation and suggest that coordinated activation of multiple effector muscles may be necessary to sustain airway patency and effectively treat OSA. Nevertheless, stimulating multiple motor nerves in OSA patients is challenging due to the mixed functions, deep anatomy, and overlapping roles of UA effector nerves.

Phrenic nerve stimulation (PNS) may offer an alternative strategy for treating OSA. The phrenic nerve is a mixed motor–sensory nerve, with efferent fibers driving diaphragmatic contraction and afferent fibers providing sensory feedback to modulate respiratory and UA muscle activity.^7^^,^^8^ PNS can be detrimental to UA stability because it can exaggerate inspiratory flow limitation and induce negative effort dependence (NED) with reductions in inspiratory flow as diaphragmatic effort increases.^9^^,^^10^ Flow limitation with NED has been observed among patients who are diaphragmatically paced and leveraged experimentally to deliberately induce UA obstruction in OSA.^11-15^ Nevertheless, prior studies have also described neuromechanical mechanisms by which PNS may actually enhance ventilation and promote UA patency. PNS may increase caudal traction on the UA, thereby improving patency.^16-22^ PNS has been shown to increase ventilatory drive via afferent nerve stimulation, which in turn can enhance UA dilator activity,^23-26^ and it may trigger the negative pressure reflex, whereby abrupt drops in airway pressure recruit genioglossus activity that protects against collapse.^27-30^ These mechanisms suggest complex and context-dependent effects of PNS on UA patency, warranting further investigation.

To investigate the effects of PNS on UA patency, we tested PNS in OSA patients undergoing drug-induced sleep endoscopy (DISE). We hypothesized that PNS can relieve UA obstruction and improve ventilation. In probing the effects of PNS during preliminary experiments, we observed that the effects on the UA may depend on the timing of the stimulation in relation to inspiration. To investigate effects of PNS timing, we (1) applied PNS at specific points in the respiratory cycle and (2) explored effects of single and sequential PNS on UA patency and ventilation.

## Methods

### Study participants

Adults (≥18 years) with OSA undergoing DISE as part of presurgical evaluation were recruited. Exclusion criteria included uncontrolled cardiovascular disease, chronic obstructive pulmonary disease, or contraindications to anesthesia. Demographic data (age, sex, body mass index [BMI], neck circumference) and baseline apnea–hypopnea index (AHI) from diagnostic sleep studies were collected. Eligibility required an AHI > 5 events/hour based on the 4% desaturation criterion or >10 events/hour based on the 3% desaturation criterion without inclusion of arousals.^31^ The trial (NCT05350332) was approved by the Emory University institutional review board, and all participants provided written informed consent.

### DISE protocol

**Figure 1:**
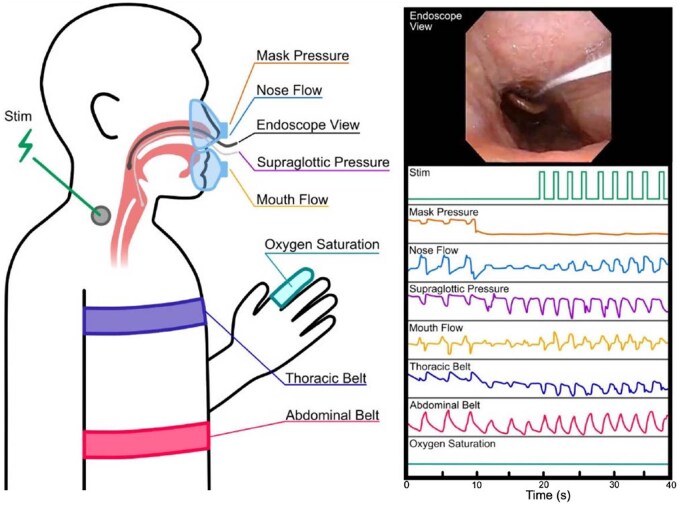
Phrenic nerve stimulation (PNS) and drug-induced sleep endoscopy (DISE) setup. A modified nasal mask equipped with an adapter enabled airway visualization via a fiber optic camera. An additional mouth mask was applied to allow separate capture of nasal and oral airflow. A pressure catheter was placed through the nasal mask and positioned just above the epiglottis. Respiratory effort belts were placed on the chest and abdomen, and a pulse oximeter was placed to monitor oxygen saturation. The stimulation electrode was positioned on the right side of the neck, and PNS strength, timing, and duration were recorded for analysis.

Participants were sedated with propofol while positioned supine. Depth of sedation was assessed using clinical signs and sedation depth monitoring (SedLine Brain Function Monitoring, Masimo, Irvine, CA, USA). Airflow, supraglottic pressure, respiratory effort, and UA endoscopy were recorded using a custom setup that allowed simultaneous CPAP titration and airway visualization (Figure 1).^32^^,^^33^

### CPAP titration

Once adequately sedated, CPAP pressure was increased stepwise to determine the critical closing pressure (P_crit_) and the pharyngeal opening pressure (P_open_).^34-36^ Following titration, CPAP was lowered to induce stable flow-limited breathing for PNS testing.

### PNS experiments

A transcutaneous electrode was placed at the right lower neck to deliver PNS.^37^ Stimulation parameters (frequency, pulse width, and amplitude) were titrated until effective diaphragm contraction was confirmed by respiratory belt motion and supraglottic pressure deflection. Two experimental paradigms were performed:

- Isolated stimulation: Single bursts were delivered at specific respiratory phases: end expiration to early inspiration (E→I), inspiration only (I→I), or late inspiration into expiration (I→E). Unstimulated breaths before and between stimulations served as controls.
- Sequential stimulation: Repeated bursts targeted to the E→I phase were applied over 4-12 consecutive breaths to examine cumulative and sustained effects. Each stimulation run included flanking control breaths.

### Analysis

Physiological data were recorded using LabChart software (version 8.1, ADInstruments Inc, Colorado Springs, CO, USA) and processed in R (R Core Team, 2025). For isolated stimulations, linear mixed-effects models (subject as random effect) assessed the effect of stimulation timing (E→I, I→I, I→E vs baseline) on peak inspiratory flow (V_I_) and tidal volume (TV) using the lmerTest package.^38^ For sequential stimulations, we modeled respiratory metrics (V_I_, TV, minute ventilation [MV], respiratory rate [RR]) across pre-, during-, and poststimulation time points. To evaluate whether effects accumulated across breaths, we also compared respiratory metric changes across the first 3 consecutive stimulations. Estimated marginal means, 95% confidence intervals, and post hoc comparisons were derived using the emmeans package,^39^ with significance set at α = 0.05. Mean supraglottic negative pressure was compared between unstimulated and stimulated breaths using a Wilcoxon signed-rank test, and the relationship between V_I_ and negative pressure changes was assessed with a Spearman rank-order correlation. Relative changes in end-expiratory lung volume (EELV) during stimulation were measured with abdominal and thoracic effort belts and were compared to unstimulated breaths with a linear mixed-effects model to account for random variability between subjects.

### Extended methods

Comprehensive details on anesthesia, instrumentation, stimulation parameters, and statistical modeling are provided in the Supplemental Methods.

## Results

### Participant characteristics

Table 1 describes patient demographics, sleep study characteristics, and DISE metrics of the patient cohort. Of 21 initial patients who were enrolled, 19 patients were included in the final full analysis with 13 patients completing isolated stimulation and 19 completing sequential stimulation (13 patients had both protocols performed). Two patients were excluded: 1 due to PNS equipment failure during DISE and 1 because poor-quality supraglottic pressure signals during the experiment prevented timing PNS to specific phases of the respiratory cycle.

**Table 1:** Demographics information of patients undergoing phrenic nerve stimulation protocols (*n* = 19).

Characteristic	Value
**Age (years)**	58.4 ± 11.37
**Sex**	
**Male**	16 (84%)
**Female**	3 (16%)
**Neck circumference (cm) (*n*** = **18)**	41.81 ± 3.18
**BMI (kg/m²)**	30.66 ± 3.08
**AHI4% (events/h) (*n*** = **16)**	31.56 ± 17.92
**Supine (*n*** = **5)**	49.50 ± 29.53
**Nonsupine (*n*** = **4)**	11.68 ± 2.47
**AHI3% (events/h) (*n*** = **12)** ^b^	40.26 ± 15.22
**Supine (*n*** = **10)**	51.68 ± 1.89
**Nonsupine (*n*** = **10)**	32.64 ± 22.14
**ODI3% (events/h) (*n*** = **7)**	36.76 ± 14.24
**ODI4% (events/h) (*n*** = **14)**	25.84 ± 17.02
**Oxygen nadir (%)**	79.68 ± 9.23
**Time spent < 88% (min)**	14.99 ± 22.72
**P_crit_ (cmH₂O) (n** = **15)**	5.04 ± 3.32
**P_open_ (cmH₂O)**	11.52 ± 4.29
**Underwent DISE with^c^**	
**Isolated stimulation sequence**	13 (68%)
**Sequential stimulation sequence**	19 (100%)
**Study type**	
**In-Lab (PSG)**	6 (32%)
**HSAT (Flow)**	1 (5%)
**HSAT (WatchPAT)**	12 (63%)

Abbreviations: AHI, apnea–hypopnea index; BMI, body mass index; DISE, drug-induced sleep endoscopy; HSAT, home sleep apnea test; ODI, oxygen desaturation index; P_crit_, critical closing pressure; P_open_, pharyngeal opening pressure; PSG, polysomnography.

a The sample size is reported for variables not available in all patients.

b Two patients were excluded from the AHI3% analysis because their sleep studies included electroencephalogram arousals (AHI3A% = 19.06, 18.9).

c Thirteen (68%) patients underwent isolated and sequential stimulation sequence.

The final study group consisted of mostly obese men with moderate-to-severe OSA. Mean P_open_ values measured during DISE were comparable to therapeutic CPAP levels reported in the natural sleep literature.^40^ In contrast, P_crit_ values were slightly higher during DISE, suggesting slightly greater airway collapsibility compared with natural sleep.^41^ Upper airway endoscopy was attempted during PNS experiments, but maintaining an adequate endoscopic view during stimulation proved technically challenging, leading to variable video quality and exclusion of video analysis.

### Isolated stimulation

**Figure 2:**
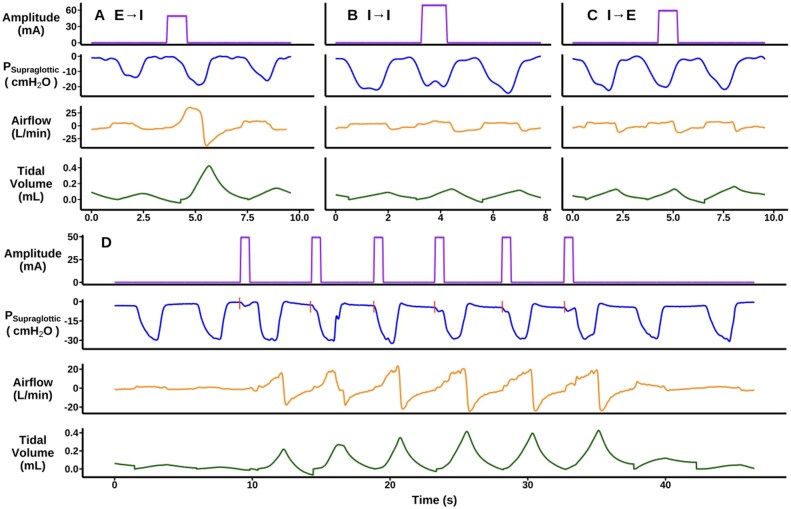
Example tracings of isolated and sequential phrenic nerve stimulus (PNS) experiments. Isolated stimulation was tested at 3 phases of respiration (A) E→I, (B) I→I, and (C) I→E. The greatest improvements in airflow and tidal volume (TV) are observed during E→I. I→I showed slight increases in airflow that were significant, while I→E stimulation did not improve airflow. Sequential stimulation (D) shows the sustained effect of PNS across multiple breaths when timed to E→I phase in each breath. Potentiation can be seen as airflow, and TV gradually increases over the first several breaths. Red ticks indicate the deflection of the Millar pressure catheter associated with PNS in these tracings. Abbreviations: E→I, end-expiration to inspiration; I→E, mid-inspiration to expiration; I→I, inspiration only.

Figure 2 A-C, shows representative tracings of the effect of PNS at different phases of inspiration. We observed greater increases of airflow, including restoration of nonflow limited breathing in some patients, during E→I (Figure 2A) stimulation. Stimulation at I→I (Figure 2B) produced marginal increases in airflow with flow-limitation remaining, while there were no significant changes to airflow with I→E (Figure 2C) stimulation.

**Figure 3:**
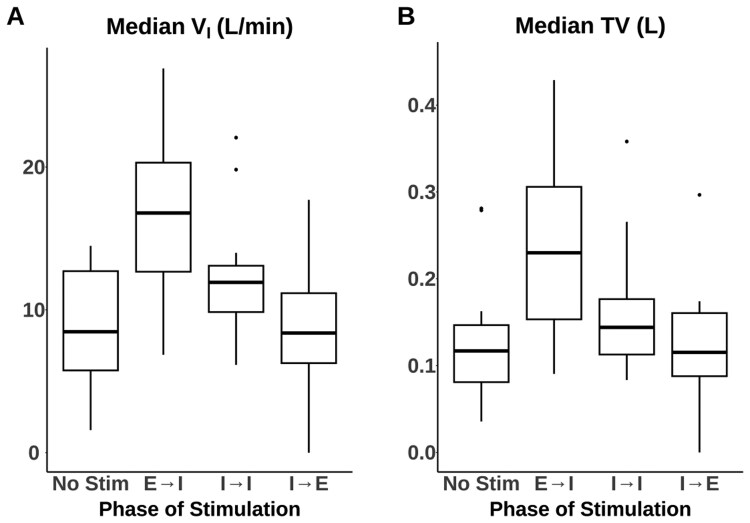
Boxplots comparing (A) inspiratory flow (V_I_) and (B) tidal volume (TV) from unstimulated baseline across the 3 phases of respiration targeted for phrenic nerve stimulation (PNS). For each phase, the data represent the median value of each patient’s sampled breaths. Significant improvements in V_I_ and TV occurred during the E→I phase of inspiration compared to stimulation timed to the other portions of inspiration. Abbreviations: E→I, end-expiration to inspiration; I→E, mid-inspiration to expiration; I→I, inspiration only.

Isolated stimulation experiments were conducted at a mean CPAP level of 6.9 cmH_2_O (±5.0 cmH_2_O), which was 4.1 cmH_2_O (95% CI, 2.5-5.7 cmH_2_O]) below P_open_. The average stimulation amplitude was 74.8 mA (±14.4 mA) and duration was 1.00 seconds (±0.19 seconds). The mixed effect analysis showed that stimulation timed to E→I resulted in a model estimated 64% increase in airflow and 71% increase in TV (Figure 3 and Table 2A) compared to unstimulated baseline breaths. Stimulation at I→I also increased V_I_ and TV but to a lesser degree (27% and 21%, respectively), while stimulation at I→E had no significant change in V_I_ but a 14% TV decrease.

**Table 2:** Model-estimated mean values for ventilatory outcome measures for both Isolated (A) and Sequential (B) stimulation protocols.

Stimulation	Estimated group means ± 95% CI			
**(A) Isolated stimulation protocol**				
**Unstimulated baseline**	10.03 ± 2.82	0.14 ± 0.04		
**E→I**	**16.49 ± 3.23^a^**	**0.24 ± 0.02^a^**		
**I→I**	**12.77 ± 3.27^a^**	**0.17 ± 0.05^a^**		
**I→E**	**9.33 ± 3.43**	**0.12 ± 0.05** ^b^		
**(B) Sequential stimulation protocol**				
**Unstimulated baseline**	6.16 ± 2.56	0.10 ± 0.05	1.84 ± 0.86	18.0 ± 2.67
**Stim. #1**	**14.88 ± 3.38^a^**	**0.21 ± 0.06^a^**	**3.54 ± 0.97^a^**	18.15 ± 0.89
**Stim. #2**	**18.04 ± 3.38^a^**	**0.28 ± 0.06^a^**	**5.00 ± 0.97^a^**	18.1 ± 0.89
**Stim. #3**	**17.33 ± 3.38^a^**	**0.30 ± 0.06^b^**	**5.23 ± 0.97^a^**	18.7 ± 0.89

(A) Model-estimated mean V_I_ and TV responses to PNS at different phases of inspiration in flow-limited OSA patients during DISE. The greatest magnitude of effect occurred in the E→I phase. (B) Model-estimated mean ventilatory metrics from sequential PNS in OSA patients during DISE showing sustained increases with continued stimulation and progressive improvement across the first three sequentially stimulated breaths, suggesting a potentiation effect.

Abbreviations: DISE, drug-induced sleep endoscopy; E→I, end-expiration to inspiration; I→E, mid-inspiration to expiration; I→I, inspiration only; MV, minute ventilation ; OSA, obstructive sleep apnea; PNS, phrenic nerve stimulation; RR, respiratory rate; Stim, stimulation; TV, tidal volume; V_I_, inspiratory flow

a Denotes significance at alpha = 0.05.

b Denotes significance at alpha = 0.01.

### Sequential stimulation

Figure 2D shows a representative tracing of the effect of PNS during repeated stimulation in the E→I phase. The airflow tracing demonstrates marked improvements in V_I_ and TV during stimulation compared with unstimulated breaths. Moreover, both V_I_ and TV show progressive increases across the first several stimulations.

**Figure 4:**
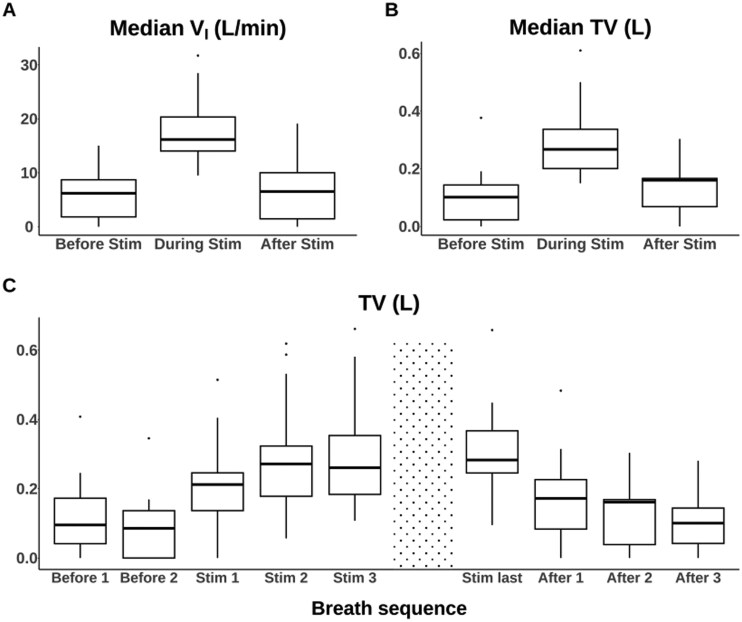
Boxplots showing the effect of sequential phrenic nerve stimulation (PNS) on (A) inspiratory flow (V_I_) and (B) tidal volume (TV) before, during, and after stimulation. For each period, the data represent the median value of each patient’s sampled breaths. Results show that V_I_ and TV increase during stimulation and return to baseline once stimulation ceases. Examining TV across sequential breaths (C) reveals a cumulative increase peaking around the third breath, suggesting potentiation of the PNS effect over multiple stimulations, potentially due to short-term potentiation or transient increases in end-expiratory lung volume (EELV). We also observed a gradual return of V_I_ and TV to baseline over several breaths, indicating a residual effect of stimulation, possibly reflecting the gradual normalization of EELV. Grayed area indicates sequential breaths between the third breath and last stimulated breath.

On average, sequential stimulation experiments were conducted at a CPAP level of 6.6 cmH_2_O (±4.8 cmH_2_O), which was 4.9 cmH_2_O (95% CI, 3.5-6.3 cmH_2_O) below P_open_. The average stimulation amplitude was 72.8 mA (±18.4 mA) and duration of 1.04 seconds (±0.24 seconds). The mixed effects analysis showed a model estimated 171% increase in V_I_, 173% increase in TV, and 169% increase in MV in stimulated breaths compared to baseline unstimulated breaths immediately prior to and after stimulation (Figure 4A and B, Table 2B). Supraglottic negative pressure did not differ significantly between stimulated and unstimulated breaths, while the Spearman rank-order correlation showed a significant association between increased airflow and reduced negative pressure (see supplementary material S1).

Additional observations were noted from the sequential stimulation protocol. Further improvements in V_I_, TV, and MV were observed across each of the first 3 stimulated breaths compared to prior unstimulated breaths (Figure 4C). Post hoc comparisons indicated further increases from the first to third sequentially stimulated breaths in V_I_ (17.33 L vs 14.88 L) and TV (0.20 L vs 0.11 L), though these were not significant (*P* > .05). Minute ventilation did increase significantly (3.39 L/min vs 1.69 L/min, *P* = .019), while respiratory rate was unchanged (see Supplementary Material S2). End-expiratory lung volume (EELV) could be estimated from thoracic and abdominal effort belts in 15 of 19 subjects (belt signals were too loose in 4 subjects). EELV increased by a model-estimated mean of 0.053 L during stimulated vs unstimulated breaths (*P* < .001). Manually triggered stimulation also occasionally produced mistimed stimulations within the sequence, which resulted in a loss of effect until a well-timed (E→I) stimulation was delivered; this phenomenon is exemplified by a mistimed sequential run shown in Supplementary Material S3. Finally, a latency of effect was observed with peak V_I_ occurring, on average, 1.41 seconds (±0.69 seconds) after PNS onset. The peak effect often also occurred after the end of a stimulation burst, with a latency averaging 0.36 seconds after stimulation ended (±0.75 seconds).

## Discussion

We found that transcutaneous PNS increases inspiratory flow and ventilation during flow-limited breathing under propofol sedation and that the response depended on the timing of PNS within the respiratory cycle. Specifically, E→I showed the greatest increases with lesser improvements during I→I and slight worsening of TV during E→I. In addition, sequential stimulation at E→I showed sustained improvements in airflow and ventilation (V_I_, TV, MV, RR) including potentiation over several consecutive stimulations. PNS responses also exhibited a latency, with airflow peaking beyond the end of the stimulation burst. Our findings indicate that PNS enhances UA patency and restores ventilation. In the discussion below, we examine possible neuromechanical mechanisms for PNS responses and potential therapeutic implications for OSA patients.

Our findings add to the body of literature that suggests PNS can promote airway patency rather than induce collapse.^42^ Typically, when increasingly negative pressure is generated by diaphragmatic contraction during sleep or sedation, inspiratory airflow limits at a maximal level that is not exceeded despite further reductions in supraglottic pressure.^43^ In fact, rather than generating additional flow, increasing negative pressure across a collapsible (flow-limited) airway can actually decrease airflow below the maximal inspiratory flow level, a phenomenon described generally in collapsible biologic conduits as NED.^9^^,^^10^ In contrast, we found that V_I_ actually increased during PNS, indicating some degree of lessening airflow obstruction. Increases in V_I_ were correlated with reductions in supraglottic negative pressure, also suggesting that the airway obstruction was lessened. Concurrent increases in TV suggest that PNS can stabilize UA patency and ventilation during sedation. Increases in TV were also attributed to concomitant PNS-induced decreases in NED in some patients (see Supplementary Material S4), further supporting that stimulation can enhance ventilation without inducing airway collapse. The observed increases in airflow and ventilation alongside reductions in supraglottic negative pressure suggest that PNS favorably modifies pressure–flow dynamics in a collapsible airway.

Several findings implicate neural mechanisms mediating PNS responses in our study. First, PNS responses were timing-dependent, with stimulation at end-expiration (E→I) yielding the greatest increases in flow and ventilation. Second, airflow responses lagged PNS bursts, peaking more than a second after the PNS onset, suggesting intermediate neural processing of PNS-induced reflex responses. Third, airflow responses to PNS were maintained throughout the inspiratory phase, peaking after the end of the stimulation burst, suggesting that PNS engaged intrinsic generators of phasic UA neuromotor activity. Evidence from the literature suggests the importance of coordinating UA muscle activity at end-inspiration with next inspiration. In healthy individuals during sleep, UA dilator muscles activate just before inspiration, peaking before diaphragmatic contraction.^44^ In contrast, in OSA, this neuromuscular coordination deteriorates: dilator activation becomes progressively delayed as airway obstruction rises across breaths, eventually lagging behind inspiratory effort.^45^ As noted earlier, airway dilators do respond to abrupt negative pressure (negative pressure reflex),^27-30^ and stimulation of phrenic nerve afferents at end-expiration may reset reflex coordination of respiratory effort and UA dilators. The pattern of this response stands in sharp contrast to that observed during hypoglossal stimulation. The latter leads to an abrupt onset and offset of airflow responses that coincide temporally with the stimulation burst due to direct muscular activation.^46^ The prolonged latency and persistence of PNS airflow responses suggest that signals from phrenic afferents and airway mechanoreceptors mediate the observed increases in pharyngeal stability and ventilation.

Progressive breath-to-breath increases in airflow during sequential PNS suggest additional mechanisms that further amplify PNS responses to isolated stimulation. Prior studies have established that PNS can evoke short-term potentiation of inspiratory phrenic nerve activity, whereby repeated neural stimulation amplifies neuromuscular responses.^47^ Alternatively, sequential PNS may increase EELV to augment caudal traction on the UA and reduce UA collapsibility.^16-22^ In our study, mechanical effects of EELV on UA patency were variably observed with a progressive rise in thoracic volume during sequential stimulation that gradually returned to baseline after stimulation (see Supplementary Material S3). Gradual changes in EELV associated with sequential PNS suggest that the lungs hyperinflate dynamically when consecutive breaths were stimulated.^22^^,^^48^^,^^49^ It is possible that neural and mechanical mechanisms contribute together to enhance airflow responses to sequential PNS.

PNS was performed under propofol sedation, which has important implications for interpreting the results. Propofol sedation increases slow wave electroencephalogram (EEG) activity that is similar but not equivalent to nonrapid eye movement sleep.^50^ Propofol can suppress both ventilatory drive and UA muscle activity in a dose-dependent fashion that may not perfectly mimic the pathophysiology of UA collapse in OSA.^51^^,^^52^ Nonetheless, prior work has shown that ventilatory responses to flow-limited breathing are observed under propofol sedation, and pressure-flow relationships of the UA behave similarly to natural sleep.^33^^,^^53-56^ Collectively, these findings support the use of sedation as a model for studying UA collapse in OSA, but observations ultimately need to be confirmed in sleep.

Transcutaneous PNS stimulation could potentially induce arousals, thereby confounding interpretation of airflow improvements. Electroencephalogram analysis of arousals was not possible as the Sedline frontal EEG uses proprietary technology to produce its patient sedation index, and we are unable to review the EEG waveforms. Several observations argue against arousal as the primary mechanism underlying our findings. First, airflow improvements were tightly linked to stimulation timing within a narrow window of the inspiratory phase, whereas arousal-related responses would be expected to produce more immediate and sustained ventilatory effects, independent of phase. Second, the airflow increase during isolated stimulation was limited to a single breath, in contrast to arousal responses, which typically persist across multiple subsequent breaths. Finally, mistimed sequential stimulation (outside the E→I phase) produced no ventilatory improvement compared to well-timed stimulation (during E→I), as seen in Supplementary Material S3, suggesting that even serial stimulation did not induce arousals to increase ventilation. Although our data provide evidence that the observed effects are not driven by arousals, confirmation of these findings will require further studies conducted during sleep.

The observed responses to PNS could involve activation of adjacent, nonphrenic nerve targets by the transcutaneous electrode. Direct stimulation of pharyngeal muscles could improve airway patency independently of phrenic nerve activation. This explanation is unlikely because the concentric circumferential electrode confined stimulation to the region immediately beneath the electrode and was positioned low in the neck near the clavicle, where phrenic nerve capture is optimal.^37^ Moreover, nonspecific muscle activation would be expected to produce an immediate on/off improvement in airway patency independent of respiratory timing, similar to that of hypoglossal neurostimulation.^46^ In contrast, the responses observed in our experiments occurred only when stimulation was delivered at end-expiration and exhibited a latency to peak effect. Finally, effective phrenic nerve capture with surface electrodes was sensitive to small changes in electrode position that affected diaphragm capture and response. This sensitivity to electrode position suggests activation of a relatively small discrete target, rather than large muscles. Future studies with fine-wire electrodes will be needed to isolate and selectively stimulate the nerve to fully exclude concerns of off-target effects.

Endoscopic evaluation of PNS-related effects was not included due to variable video quality. Direct visualization of UA responses to PNS could provide additional insight into the mechanisms underlying the observed effect. For example, quantitative cross-sectional area analysis may help define the magnitude of airway dilation in response to stimulation. We plan to refine the experimental setup, including improved maintenance of endoscopic camera position, to enable these analyses in future studies.

In conclusion, this study presents novel evidence that PNS, when delivered at end-expiration, can enhance UA patency in OSA patients during DISE. Additionally, our experiments provide early insights into the possible neuromechanical mechanisms mediating responses. Further studies are needed to delineate the neural vs mechanical contributors to the observed potentiation, which may help refine PNS strategies for mitigating UA collapse during sleep in OSA patients. These results open promising new avenues for developing phrenic nerve–based neurostimulation therapies aimed at treating OSA.

## Supplementary Material

aaoag079_Supplementary_Data
