# Supplementary material for: Phrenic nerve stimulation enhances upper airway patency during drug-induced sleep endoscopy in obstructive sleep apnea
Source: Ann Am Thorac Soc. 2026 Apr 26;23(8):1207–16. doi: 10.1093/annalsats/aaoag079 (PMC13424842; doi:10.1093/annalsats/aaoag079)
Supplement: aaoag079_Supplementary_Data [file aaoag079_supplementary_data.zip › Supplement 2.docx]

**Supplement 2**: Model-estimated mean ventilatory metrics in response to PNS were analyzed across the first three stimulated breaths of each patient’s sequential stimulation run. V_I_, TV, and MV all showed significant increases compared to baseline, indicating a sustained positive ventilatory effect. Post-hoc pairwise comparisons with Tukey adjustment of TV and Vmax revealed a difference between the first and third stimulated breaths, but they were not significant at alpha=0.05. MV was significantly higher in the third stimulated breath than the first. Group mean estimates and 95% confidence intervals for the three stimulation bursts are provided.  * denotes significance at alpha=0.05, ** at alpha=0.01.

V_I:_ Model Fixed Effects Summary and posthoc pairwise test results

| effect | term | estimate | std.error | statistic | df | p.value | conf.low | conf.high | estimated_group_mean | group_mean_lower_95 | group_mean_upper_95 |
| --- | --- | --- | --- | --- | --- | --- | --- | --- | --- | --- | --- |
| fixed | (Intercept) | 6.16 | 1.31 | 4.71 | 39.62 | 0 | 3.60 | 8.73 |  |  |  |
| fixed | categoryStim1 | 8.72 | 1.73 | 5.05 | 73.00 | 0 | 5.33 | 12.10 | 14.88 | 11.60 | 18.16 |
| fixed | categoryStim2 | 11.88 | 1.73 | 6.88 | 73.00 | 0 | 8.50 | 15.26 | 18.04 | 14.76 | 21.32 |
| fixed | categoryStim3 | 11.16 | 1.73 | 6.47 | 73.00 | 0 | 7.78 | 14.54 | 17.33 | 14.05 | 20.60 |

| contrast | estimate | SE | df | t.ratio | p.value |
| --- | --- | --- | --- | --- | --- |
| Before - Stim1 | -8.716 | 1.726 | 73 | -5.050 | 0.000 |
| Before - Stim2 | -11.878 | 1.726 | 73 | -6.883 | 0.000 |
| Before - Stim3 | -11.162 | 1.726 | 73 | -6.468 | 0.000 |
| Stim1 - Stim2 | -3.162 | 1.993 | 73 | -1.587 | 0.392 |
| Stim1 - Stim3 | -2.446 | 1.993 | 73 | -1.227 | 0.612 |
| Stim2 - Stim3 | 0.716 | 1.993 | 73 | 0.359 | 0.984 |

Tidal Volume: Model fixed effects summary and posthoc pairwise test results

| effect | term | estimate | std.error | statistic | df | p.value | conf.low | conf.high | estimated_group_mean | group_mean_lower_95 | group_mean_upper_95 |
| --- | --- | --- | --- | --- | --- | --- | --- | --- | --- | --- | --- |
| fixed | (Intercept) | 0.10 | 0.02 | 4.12 | 35.18 | 0 | 0.05 | 0.15 |  |  |  |
| fixed | categoryStim1 | 0.11 | 0.03 | 3.53 | 73.00 | 0 | 0.05 | 0.16 | 0.21 | 0.15 | 0.27 |
| fixed | categoryStim2 | 0.18 | 0.03 | 6.16 | 73.00 | 0 | 0.13 | 0.24 | 0.28 | 0.22 | 0.34 |
| fixed | categoryStim3 | 0.20 | 0.03 | 6.54 | 73.00 | 0 | 0.14 | 0.25 | 0.30 | 0.24 | 0.36 |

| contrast | estimate | SE | df | t.ratio | p.value |
| --- | --- | --- | --- | --- | --- |
| Before - Stim1 | -0.105 | 0.030 | 73 | -3.529 | 0.004 |
| Before - Stim2 | -0.184 | 0.030 | 73 | -6.160 | 0.000 |
| Before - Stim3 | -0.195 | 0.030 | 73 | -6.543 | 0.000 |
| Stim1 - Stim2 | -0.079 | 0.034 | 73 | -2.279 | 0.113 |
| Stim1 - Stim3 | -0.090 | 0.034 | 73 | -2.610 | 0.052 |
| Stim2 - Stim3 | -0.011 | 0.034 | 73 | -0.332 | 0.987 |

Minute Ventilation: Model fixed effects summary and posthoc pairwise test results

| effect | term | estimate | std.error | statistic | df | p.value | conf.low | conf.high | estimated_group_mean | group_mean_lower_95 | group_mean_upper_95 |
| --- | --- | --- | --- | --- | --- | --- | --- | --- | --- | --- | --- |
| fixed | (Intercept) | 1.84 | 0.44 | 4.19 | 31.91 | 0 | 0.98 | 2.71 |  |  |  |
| fixed | categoryStim1 | 1.69 | 0.49 | 3.44 | 72.16 | 0 | 0.73 | 2.66 | 3.54 | 2.49 | 4.58 |
| fixed | categoryStim2 | 3.16 | 0.49 | 6.40 | 72.16 | 0 | 2.19 | 4.12 | 5.00 | 3.96 | 6.04 |
| fixed | categoryStim3 | 3.39 | 0.49 | 6.88 | 72.16 | 0 | 2.42 | 4.36 | 5.23 | 4.19 | 6.28 |

| contrast | estimate | SE | df | t.ratio | p.value |
| --- | --- | --- | --- | --- | --- |
| Before - Stim1 | -1.695 | 0.493 | 72.080 | -3.438 | 0.005 |
| Before - Stim2 | -3.157 | 0.493 | 72.080 | -6.404 | 0.000 |
| Before - Stim3 | -3.390 | 0.493 | 72.080 | -6.876 | 0.000 |
| Stim1 - Stim2 | -1.463 | 0.566 | 72.003 | -2.583 | 0.056 |
| Stim1 - Stim3 | -1.695 | 0.566 | 72.003 | -2.994 | 0.019 |
| Stim2 - Stim3 | -0.233 | 0.566 | 72.003 | -0.411 | 0.976 |

| effect | term | estimate | std.error | statistic | df | p.value | conf.low | conf.high | estimated_group_mean | group_mean_lower_95 | group_mean_upper_95 |
| --- | --- | --- | --- | --- | --- | --- | --- | --- | --- | --- | --- |
| fixed | (Intercept) | 17.99 | 1.19 | 15.11 | 19.13 | 0.00 | 15.66 | 20.32 |  |  |  |
| fixed | categoryStim1 | 0.06 | 0.45 | 0.14 | 72.02 | 0.89 | -0.83 | 0.95 | 18.05 | 15.52 | 20.59 |
| fixed | categoryStim2 | 0.40 | 0.45 | 0.89 | 72.02 | 0.38 | -0.49 | 1.29 | 18.39 | 15.86 | 20.92 |
| fixed | categoryStim3 | 0.75 | 0.45 | 1.66 | 72.02 | 0.10 | -0.14 | 1.64 | 18.74 | 16.21 | 21.28 |

| contrast | estimate | SE | df | t.ratio | p.value |
| --- | --- | --- | --- | --- | --- |
| Before - Stim1 | -0.063 | 0.453 | 72.008 | -0.138 | 0.999 |
| Before - Stim2 | -0.401 | 0.453 | 72.008 | -0.885 | 0.813 |
| Before - Stim3 | -0.752 | 0.453 | 72.008 | -1.659 | 0.353 |
| Stim1 - Stim2 | -0.339 | 0.520 | 72.000 | -0.651 | 0.915 |
| Stim1 - Stim3 | -0.689 | 0.520 | 72.000 | -1.324 | 0.551 |
| Stim2 - Stim3 | -0.351 | 0.520 | 72.000 | -0.674 | 0.907 |
