# Supplementary material for: Phrenic nerve stimulation enhances upper airway patency during drug-induced sleep endoscopy in obstructive sleep apnea
Source: Ann Am Thorac Soc. 2026 Apr 26;23(8):1207–16. doi: 10.1093/annalsats/aaoag079 (PMC13424842; doi:10.1093/annalsats/aaoag079)
Supplement: aaoag079_Supplementary_Data [file aaoag079_supplementary_data.zip › Supplemental Methods.docx]

*Study Participants*

Eligible participants were adults (≥18 years) with OSA who underwent DISE as part of their pre-surgical evaluation for sleep apnea surgery. Exclusion criteria included uncontrolled hypertension, unstable angina, uncompensated heart failure, chronic obstructive pulmonary disease, or any other contraindication to anesthesia or surgery. Collected demographic data included age, sex, body mass index (BMI), and neck circumference. Baseline apnea-hypopnea index (AHI) severity and characteristics were obtained from clinical diagnostic sleep studies, including both in-laboratory polysomnography and home sleep apnea testing. Hypopneas were reported using either 3% or 4% desaturation criteria—or both—depending on the parameters provided in each study report.^31^ Inclusion required an AHI >5 events/hour (4% criteria) or >10 events/hour (3% criteria). This prospective clinical trial (NCT05350332, clinicaltrials.gov) was approved by the Emory University Institutional Review Board (IRB STUDY00003579) and all participants signed informed consent.

*DISE protocol*

*Anesthesia:* At the start of the DISE procedure, patients were positioned supine on a stretcher, and our recording instruments were placed (**Figure 1**). Glycopyrrolate (0.2 mg) was administered at the start of sedation to reduce pharyngeal secretions. The initial propofol infusion rate was set between 75-125 mcg/kg/min and increased by 25 mcg/kg/min every 2-4 minutes until sedation and UA flow limitation were observed. Optional 10mg boluses were also applied to maintain sedation as needed. The depth of sedation was assessed based on clinical signs, such as unresponsiveness to verbal commands, snoring, or intermittent airway collapse, in addition to a patient sedation index (PSI) of 20-60, monitored via brain function monitoring (SedLine® Brain Function Monitoring, Masimo, Irvine, CA).

*Instrumentation:* Respiratory effort was tracked using thoracic and abdominal effort belts, as well as using a pressure catheter (Mikro-Cath™, Millar, Houston, TX) that was inserted through one nostril and positioned in the supraglottic airway. Airflow was measured with a calibrated pneumotachometer (SFM3300-250-AW, Sensirion AG, Stäfa, Switzerland) connected to a customized nasal mask.^32,33^ This mask included a port for endoscopic airway visualization with a fiber-optic camera and allowed CPAP titration. A separate mouth-only mask, fitted with a second pneumotachometer, was used to monitor oral airflow. All data were captured and annotated using a PowerLab 16/35 Data Acquisition System with LabChart software (version 8.1, ADInstruments Inc, Colorado Springs, CO).

*Continuous Positive Airway Pressure (CPAP) Titration*

A CPAP unit was connected to the nasal mask, and titration began at 3–4 cmH₂O, increasing nasal pressure step-wise by 1 cmH₂O every 6–8 breaths to determine: **(a)** the highest nasal pressure associated with complete airway occlusion (critical closing pressure, P_crit_), and **(b)** the pressure required to eliminate inspiratory flow limitation (pharyngeal opening pressure, P_open_).^34–36^ CPAP was then reduced from P_open_ to a level that induced stable, flow-limited breathing for PNS testing.

*PNS Experiments*

*PNS setup and approach:* A concentric transcutaneous electrode was positioned on the right side of the neck, just above the clavicle, along the posterior border of the sternocleidomastoid muscle. This placement was informed by previous studies demonstrating it as an effective site for transcutaneous PNS.^37^ Stimulation was delivered using a DS8R Biphasic Constant Current Stimulator (Digitimer, Fort Lauderdale, FL). Stimulation was manually given by pressing a trigger button. Manual pressure was applied to the electrode to ensure proper contact and to mitigate collateral brachial plexus stimulation, which in some patients caused involuntary right arm movements. Stimulation parameters included a frequency of 30hz, pulse width of between 200-400µs and current amplitudes ranging from 40 to 100 mA. Stimulation duration, set between 0.5 and 2.0 seconds, was tailored to each patient's respiratory rate and inspiratory time. We performed test stimulations and adjusted parameters until we confirmed effective phrenic nerve stimulation, defined by physiological observations including paradoxical motion of the thoracic and abdominal respiratory belts, and deflection in the supraglottic pressure catheter during stimulation. Once the settings were confirmed, we proceeded with our experimental protocols.

*PNS protocol:* The PNS paradigm was designed to examine responses in UA patency at specific phase of the respiratory cycle. Two protocols were elaborated to examine the impact of differential PNS timing and whether the effects could be sustained as follows.

1. **Isolated stimulation**: Single stimulation bursts were delivered at specific phases of the respiratory cycle. These included:
   - **E→I**: Stimulation initiated at end-expiration and continued into early inspiration.
   - **I→I**: Stimulation both started and ended within the inspiratory phase.
   - **I→E**: Stimulation began after mid-inspiration and extended into expiration.

The timing of inspiration was determined using respiratory effort signals from the supraglottic pressure catheter. Unstimulated breaths which occurred for two breaths prior to the first stimulation and unstimulated breaths between stimulations were considered control breaths.

1. **Sequential stimulation**: Having observed PNS responses were greatest at end-expiration to inspiration (**E→I**), we targeted PNS to this phase over 4–12 consecutive breaths to assess sustained effects on the airway. Each stimulation run was bracketed by 2–3 unstimulated control breaths.

*Analysis*

All data processing and analyses were performed in R (R Core Team, 2025). To account for within-subject correlation of observations, we fit linear mixed effects models with subject as a random effect using the lmerTest package.^38^ For the *isolated stimulation protocol*, we estimated the effect of stimulation timing (independent variable with four levels: **E→I, I→I, I→E,** and unstimulated baseline) on two dependent variables: inspiratory flow (V_I_) and tidal volume (TV). Total V_I_ and TV were calculated by summing mouth and nasal mask values. For the *sequential stimulation protocol*, we estimated the effect of stimulation (independent variable with three fixed time points: before, during, or after stimulation) on V_I_, TV, minute ventilation (MV), and respiratory rate (RR).

During the *sequential stimulation protocol* we noticed that successive stimulations sometimes produced further increases in V_I_ and TV. To determine whether stimulation effects were potentiated across successive stimulations, we examined changes in respiratory metrics over the first three stimulations of the sequential run. We fit a model with a single independent variable with four levels: first, second, and third stimulation, and unstimulated baseline. Using the emmeans package^39^, we estimated model-predicted group means, 95% confidence intervals, and post-hoc pairwise comparisons with a Tukey adjustment.^50^ Statistical significance was inferred at alpha=0.05 unless otherwise noted.
