# Supplementary figures and images for: Phrenic nerve stimulation enhances upper airway patency during drug-induced sleep endoscopy in obstructive sleep apnea
Source: Ann Am Thorac Soc. 2026 Apr 26;23(8):1207–16. doi: 10.1093/annalsats/aaoag079 (PMC13424842; doi:10.1093/annalsats/aaoag079)

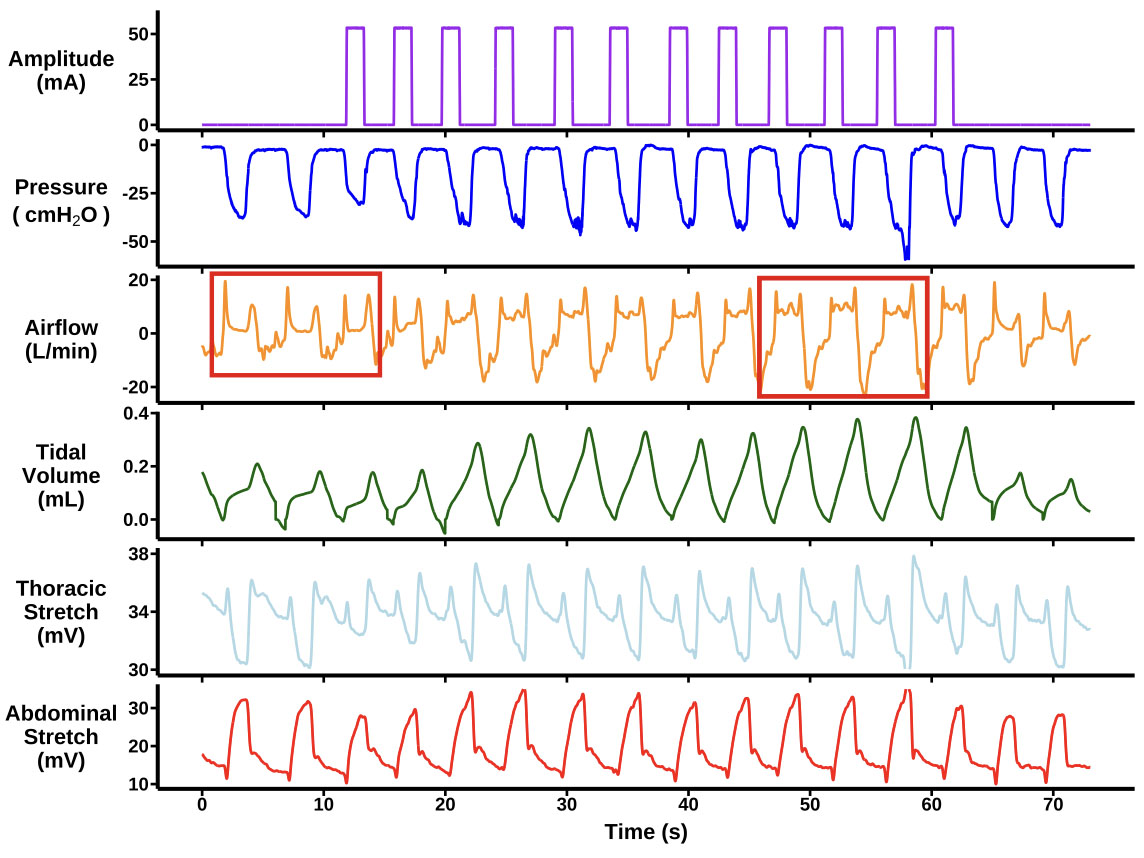

Supplement: aaoag079_Supplementary_Data [file aaoag079_supplementary_data.zip › Supplement 4.jpeg]

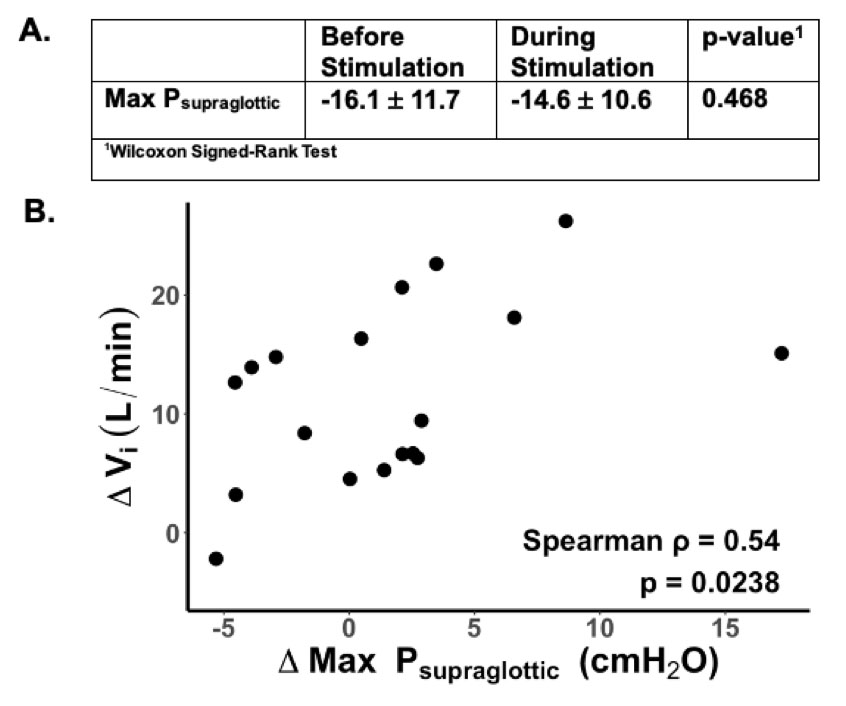

Supplement: aaoag079_Supplementary_Data [file aaoag079_supplementary_data.zip › Supplement 1.jpeg]

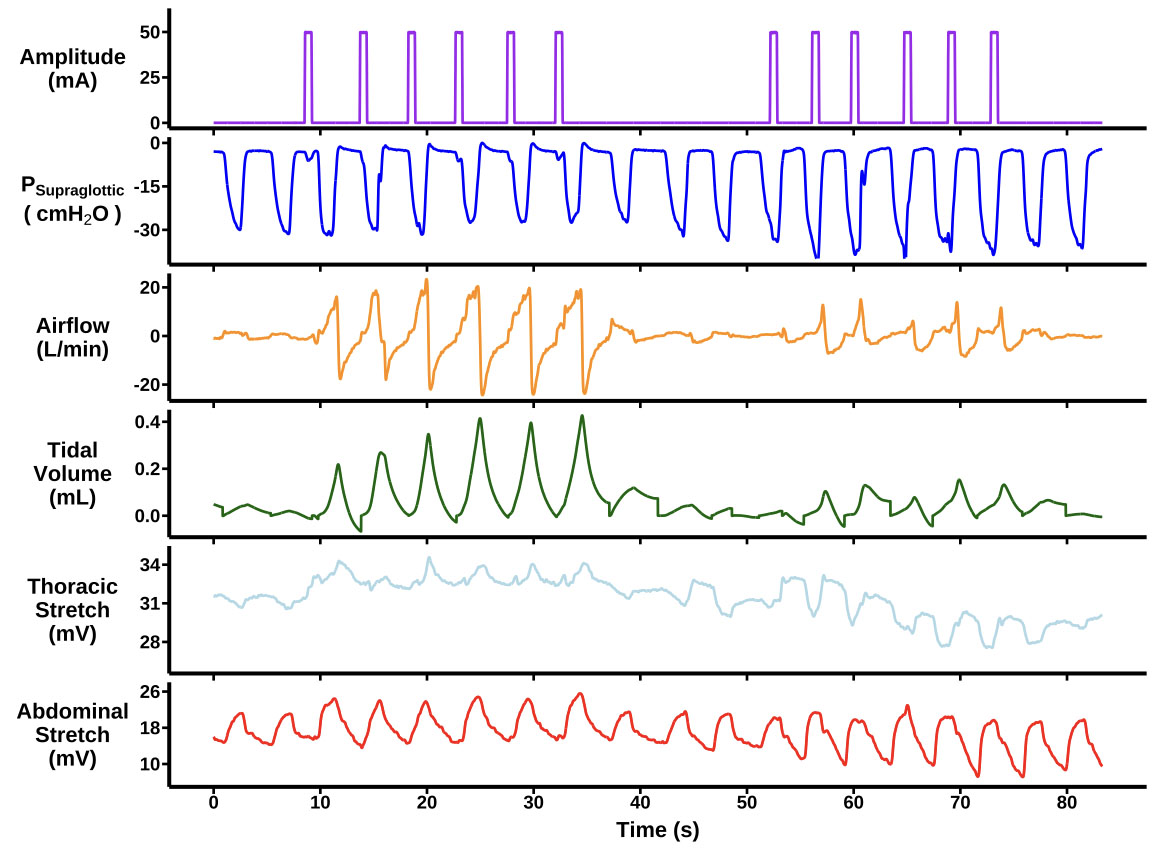

Supplement: aaoag079_Supplementary_Data [file aaoag079_supplementary_data.zip › Supplement 3.jpeg]
